# Vertical-Strip-Fed Broadband Circularly Polarized Dielectric Resonator Antenna

**DOI:** 10.3390/s17081911

**Published:** 2017-08-18

**Authors:** Amir Altaf, Jin-Woo Jung, Youngoo Yang, Kang-Yoon Lee, Keum Cheol Hwang

**Affiliations:** 1 Division of Electronics and Electrical Engineering, Dongguk University, Seoul 100-715, Korea; amiraltaf@dongguk.edu (A.A.); jinwjung@dongguk.edu (J.-W.J.); 2 School of Electronic and Electrical Engineering, Sungkyunkwan University, Suwon 440-746, Korea; yang09@skku.edu (Y.Y.); klee@skku.edu (K.-Y.L.)

**Keywords:** broadband antenna, circular polarization, dielectric resonator antenna, vertical-strip feeding

## Abstract

A vertical-strip-fed dielectric resonator antenna exhibiting broadband circular polarization characteristics is presented. A broad 3 dB axial ratio bandwidth (ARBW) is achieved by combining multiple orthogonal modes due to the use of a special-shaped dielectric resonator. The proposed antenna is fabricated to evaluate its actual performance capabilities. The antenna exhibits a measured 3 dB ARBW of 44.2% (3.35–5.25 GHz), lying within a −10 dB reflection bandwidth of 82.7% (2.44–5.88 GHz). The measured peak gain within 3 dB ARBW is found to be 5.66 dBic at 4.8 GHz. The measured results are in good agreement with the simulated results.

## 1. Introduction

The rapid growth of wireless communication systems has increased the demand for high-performance antennas. Due to their minimal conduction losses, dielectric resonator antennas (DRAs) have gained preference over metallic antennas. Specifically, DRAs with circularly polarized (CP) radiation have received more attention in relation to such systems, owing to additional advantages such as better mobility, minimal polarization loss, and the mitigation of multipath interference as compared to linearly polarized DRAs [1].

In CP DRAs, circular polarization is obtained by exciting two orthogonal modes of equal magnitude, which can be achieved by either a single-point or a dual-point feeding technique. The former technique has taken precedence over the latter given its simplicity and compactness, but the 3 dB axial ratio bandwidth (ARBW) is typically 3–6% [2,3]. Therefore, designing single-point fed CP DRAs with a broad 3 dB ARBW has become an interesting area of research. Recently, one method was developed which combines CP bands from a coupling slot and dielectric resonator (DR)—known as a hybrid antenna—to obtain a broad 3 dB ARBW [4,5,6]. For instance, the Spidron fractal dielectric resonator with a C-shaped slot is designed to produce a 3 dB ARBW of 11.57% [4]. Similarly, a 3 dB ARBW of 23.75% was achieved by combining a Spidron fractal slot antenna and a grooved DR [5]. In one study [6], the combination of a modified cross-slot and a rectangular DR yielded a 3 dB ARBW of 24.6%. Another method involved the design of special-shaped DRs which by virtue of their shape produced a broad 3 dB ARBW while using simple feeding technique [7,8,9]. For example, a pixelated DR fed through a rectangular slot was presented, demonstrating a 3 dB ARBW of 14.63% [7]. In another study [8], a multiple-circular-sector DR excited through a rectangular slot exhibited a 3 dB ARBW of 19.3%. A trapezoidal DR excited through an inclined rectangular slot attained a 3 dB ARBW of 21.5% [9]. It is important to note that the full ground plane was utilized in these methods. Unlike the aforementioned methods, DRAs with a partial ground plane have also been developed recently with a compact size and a broad 3 dB ARBW [10,11]. In contrast to the full ground plane configuration, the partial ground plane in these antennas acts as a radiator along with the DR, due to which the reflection coefficient and the 3 dB ARBW of the antenna are sensitive to the fields on the partial ground plane. By choosing the optimum dimensions of the ground plane, a broad 3 dB ARBW can be obtained. For instance, a cubic DR excited with a modified microstrip feed achieves a 3 dB ARBW of 20.6% [10]. Mohsen et al. presented a design which utilizes a square DR with two unequal inclined slits excited by a vertical strip and an L-shaped partial ground plane [11] having an overlapping bandwidth (3 dB ARBW lying within a −10 dB reflection bandwidth) of 36%.

In this paper, a simple DRA with small vertical-strip excitation is proposed. The antenna is designed with a partial ground plane. To validate the performance of the proposed DR, a fair comparison with a rectangular DR of equivalent relative permittivity is carried out using the same simulation model. It is concluded that the − 10 dB reflection bandwidths of the two DRs are nearly identical, independent of the shape of the DR. However, a broad 3 dB ARBW is obtained only in the case of the proposed DR, suggesting that multiple orthogonal modes are merged due to the specially shaped DR. All simulations are performed using the ANSYS High-Frequency Structure Simulator (HFSS) software. Details of the antenna design, parametric analysis, and measurement results are shown in the subsequent sections.

## 2. Antenna Design

**Figure 1:**
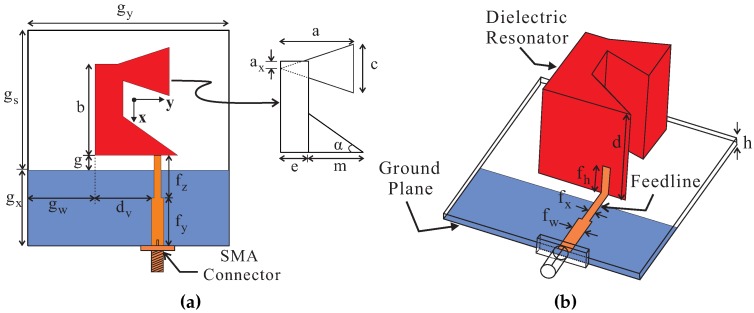
Geometry of the proposed antenna: (**a**) top view; (**b**) panoramic view. SMA: SubMiniature version A.

Figure 1 shows the geometry of the proposed antenna. The DR has height d and consists of two triangular DRs and a rectangular DR made of a ceramic material with a relative dielectric constant $\varepsilon_{dr}$ of 10 and tangent loss of 0.0002. A triangular DR with length m and angle α is connected to the lower side of the rectangular DR with length *b* and width *e*. An isosceles triangular DR of base length c and perpendicular a is connected at a distance of $a_{x}$ from the upper left corner of the rectangular DR. A partial ground plane of dimensions $g_{x}\times g_{y}$ is printed on the bottom side of an RF-35 substrate having a dielectric constant of 3.5 and dimensions $\left(g_{x}+g_{s}\right)\times g_{y}\times h$. A microstrip feedline is at the upper side of the substrate and is displaced by distance $g_{w}+d_{v}$ from the left side of the substrate. The feedline has a 50-Ω starting portion with length $f_{y}$ and width $f_{w}$. The remaining portion of width $f_{x}$ and length $f_{z}$ acts as an impedance transformer to provide wide impedance matching. A strip of width $f_{x}$ and length $f_{h}$ is mounted vertically to the wall of the DR for excitation. The DR is placed at a distance of $g_{w}$ from the left side of the substrate, while the distance between the DR and the ground plane is g.

**Figure 2:**
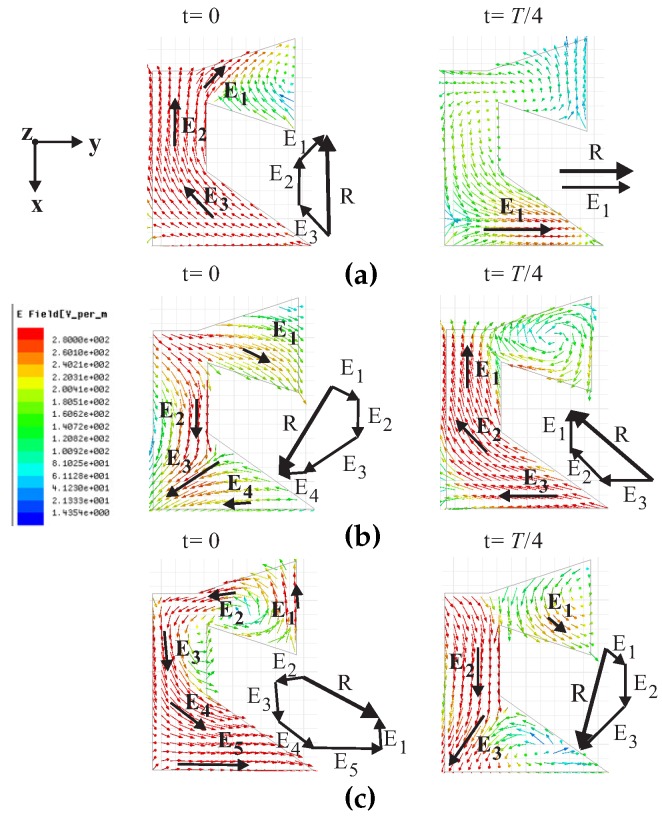
Simulated electric field distributions in a dielectric resonator (DR) with time period *T* at frequencies of (**a**) 3.5 GHz; (**b**) 4.25 GHz; and (**c**) 5 GHz.

Figure 2 shows the simulated electric field distributions in the DR as observed from the positive *z*-axis at 3.5 GHz, 4.25 GHz, and 5 GHz. Vector *E* represents the major electric field, and the vector sum is represented by *R*. For t = 0 at 3.5 GHz, in Figure 2a, the vector sum *R* is pointing towards the negative *x*-axis. For t = *T*/4, at the same frequency, the electric field near the feeding point is strongest. Hence, vector *R* rotates in the clockwise direction and becomes parallel to the *y*-axis. Similarly, the vector sum *R* for t = 0 in Figure 2b,c is rotated clockwise as well as orthogonal to *R* for t = *T*/4 at the corresponding frequencies. Therefore, the proposed antenna exhibits left-handed circular polarization (LHCP) radiation. 

## 3. Parametric Analysis

A parametric analysis was carried out to investigate the effect of varying DR length b and ground plane width $g_{x}$ on a −10 dB reflection bandwidth and a 3 dB ARBW. It should be noted that only the desired parameter was varied during the simulation.

**Figure 3:**
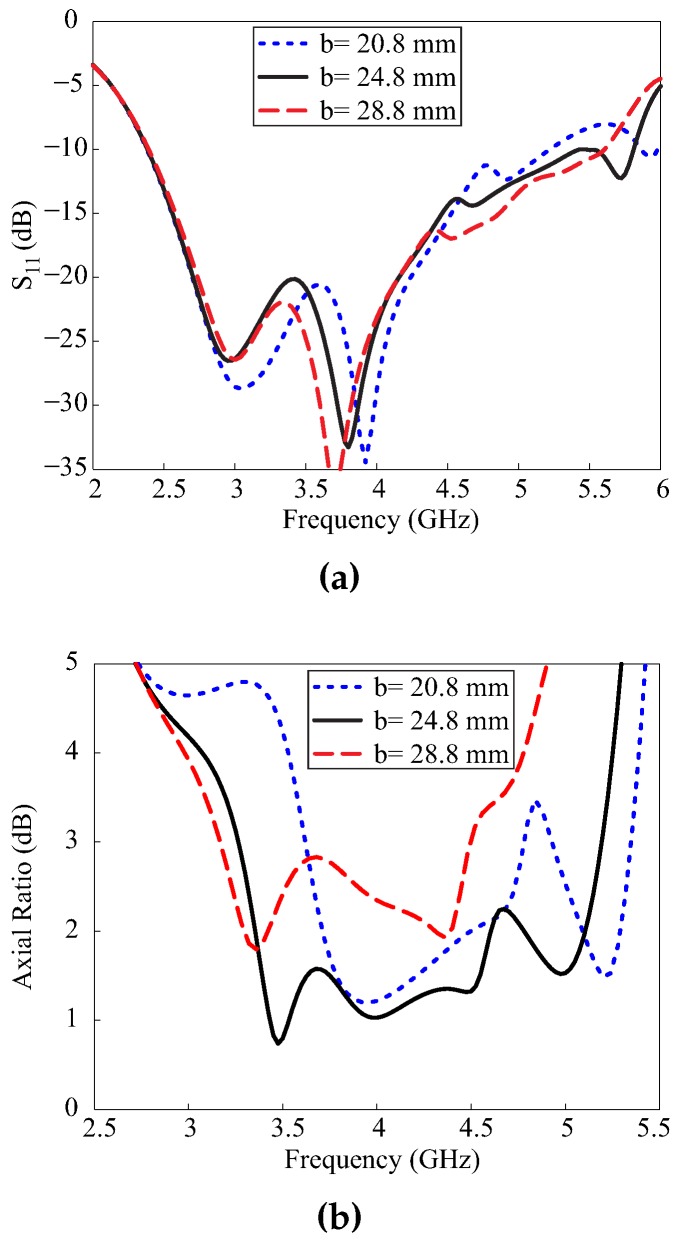
Effect of the variation of the dielectric resonator length b: (**a**) reflection coefficient; (**b**) axial ratio.

The effect of the variation of DR length *b* on the −10 dB reflection bandwidth and the 3 dB ARBW is shown in Figure 3. It was noted that the variation in length b was towards the negative x-axis with no effect on distance *g*. Figure 3a shows that the −10 dB reflection bandwidth remained mostly unaffected by these variations. On the other hand, the 3 dB ARBW moved towards lower frequencies with an increase in *b*, as shown in Figure 3b. For b = 20.8 mm, a dual-band response was observed when the axial ratio (AR) level exceeded 3 dB around 4.8 GHz. By increasing the value to b = 24.8 mm, a broad 3 dB ARBW was obtained, and a further increase in b narrowed the 3 dB ARBW.

**Figure 4:**
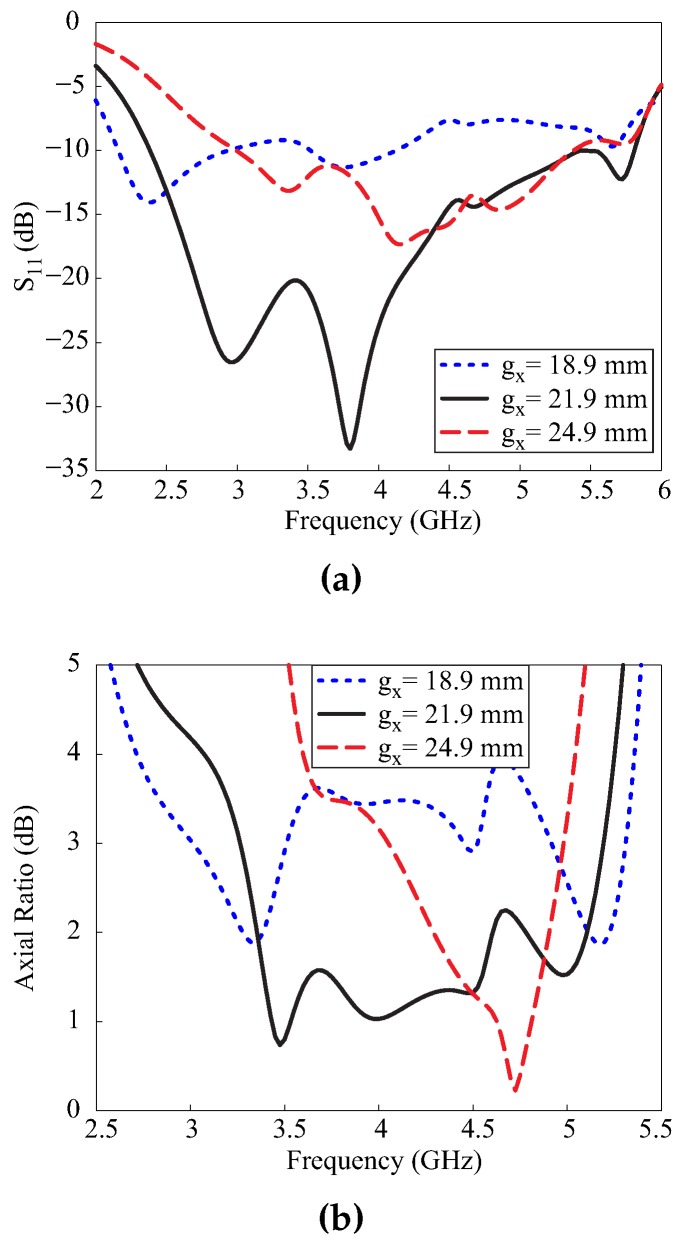
Effect of the variation of the ground plane width $g_{x}$: (**a**) reflection coefficient; (**b**) axial ratio.

Figure 4 depicts the effect of the variation of the ground plane width $g_{x}$ on the −10 dB reflection bandwidth and the 3 dB ARBW. From Figure 4a, it can be seen that the −10 dB reflection bandwidth changed due to the variation of $g_{x}$. For $g_{x}$ = 18.9 mm, the antenna had a narrow −10 dB reflection bandwidth. The widest −10 dB reflection bandwidth was obtained when $g_{x}$ = 21.9 mm, whereas it decreased again when $g_{x}$ = 24.9 mm. In Figure 4b, it can be observed that when $g_{x}$ = 18.9 mm, a dual-band CP response was obtained; however, only the lower band around 2.5 GHz laid within the −10 dB reflection bandwidth. For $g_{x}$ = 21.9 mm, the multiple orthogonal modes came closer and were merged to yield a broad 3 dB ARBW. A further increase in the value of $g_{x}$ decreased the 3 dB ARBW. Therefore, the chosen value was $g_{x}$ = 21.9 mm. Based on the parametric analysis, the optimized values are summarized in Table 1.

**Figure 5:**
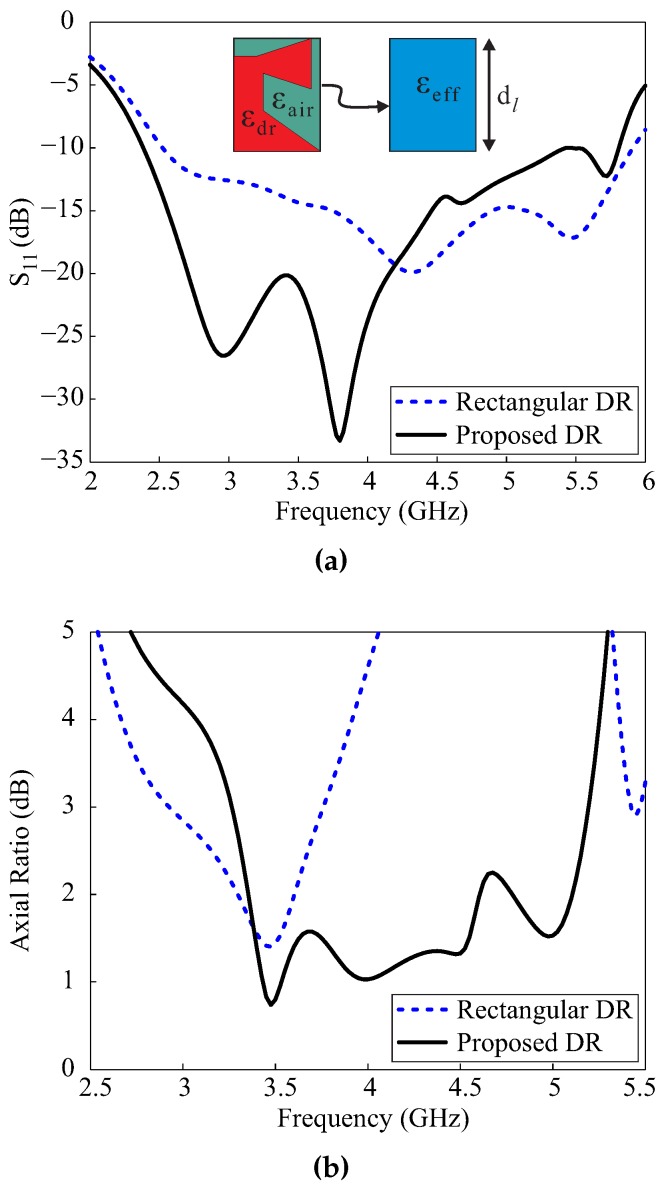
Comparison between the proposed and a rectangular dielectric resonator: (**a**) reflection coefficients; (**b**) axial ratios.

To validate the performance of the proposed design, a comparison with a rectangular DR was conducted in terms of −10 dB reflection bandwidths and ARs, as shown in Figure 5. The width and height of the rectangular DR were identical to those of the proposed DR, while the length $d_{l}$ was kept equal to the net length of the proposed DR along the *x*-axis. It is well known that the resonant frequency of a DR depends upon its volume and relative permittivity. As the volume of the rectangular DR was greater than the proposed DR, the relative permittivity required adjustment for a fair comparison. The effective relative permittivity can be calculated by a simple static capacitance model [12]:

(1) $$\varepsilon_{eff}\;=\frac{\varepsilon_{dr}\times V_{dr}+\varepsilon_{air}\times V_{air}}{V_{dr}+V_{air}},$$

where $\varepsilon_{air}$ = 1 and where the calculated value of $\varepsilon_{eff}$ is 5.77. The method of transforming the proposed DR into a rectangular DR of relative permittivity $\varepsilon_{eff}$ and length$\;d_{l}$ = 29.4 mm is visualized in Figure 5a. The results for the rectangular DR were obtained using the same simulation model shown in Figure 1, except that the proposed DR was replaced by the rectangular DR. It can also be seen in Figure 5a that the −10 dB reflection bandwidths of the proposed and rectangular DR were almost equal. The comparison of ARs is depicted in Figure 5b, which provides evidence that the broad 3 dB ARBW in the proposed antenna is attributed to the specially shaped DR.

## 4. Measurement Results and Discussion

**Figure 6:**
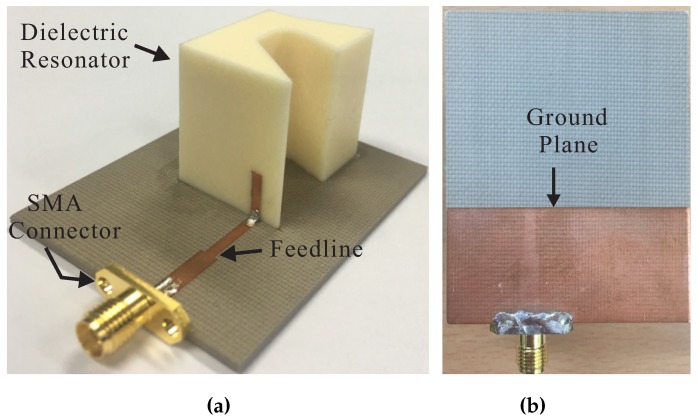
Photograph of the proposed antenna: (**a**) panoramic view; (**b**) bottom view.

**Figure 7:**
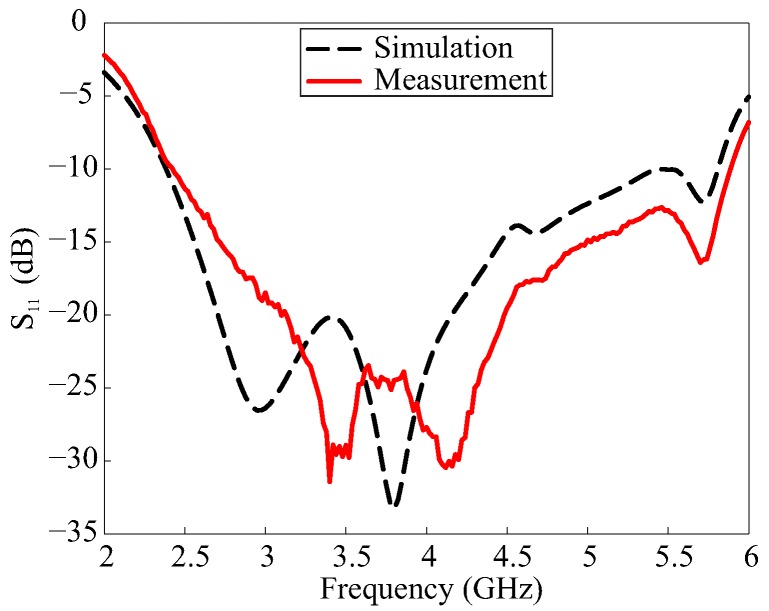
Simulated and measured reflection coefficients of the proposed antenna.

**Figure 8:**
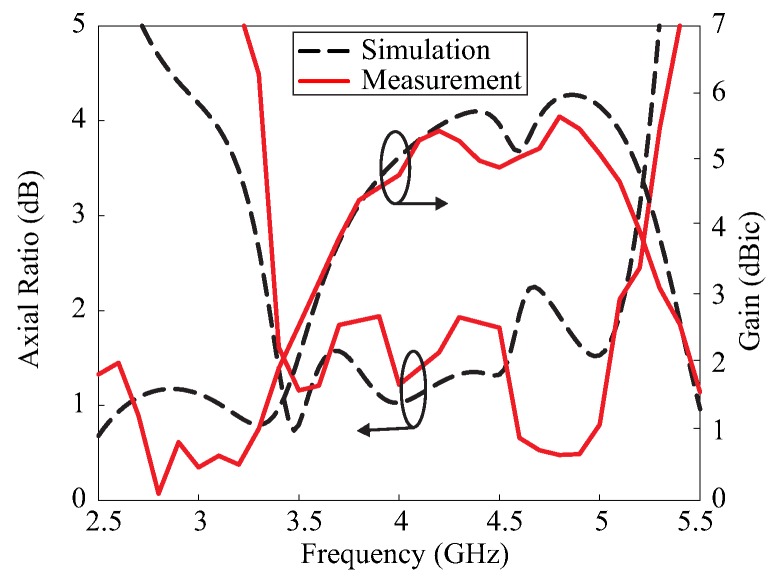
Simulated and measured axial ratios and left-handed circular polarization (LHCP) gains of the proposed antenna.

Figure 6 shows a photograph of the fabricated antenna using the optimum parameters listed in Table 1. The reflection coefficient was measured using an Agilent 8510C network analyzer and the comparison with the simulated result is presented in Figure 7. The simulated and measured −10 dB reflection bandwidths were 82.9% (2.4–5.8 GHz) and 82.7% (2.44–5.88 GHz), respectively. The far-field measurement was conducted in an RF anechoic chamber, which utilizes a dual-polarized horn antenna to assess the AR and right-handed circular polarization (RHCP)/LHCP radiation. The comparison of the simulated and measured 3 dB ARs and LHCP gains—in the broadside direction (θ = 0°)—is depicted in Figure 8. The simulated and measured 3 dB ARBWs corresponded to 45.9% (3.26–5.20 GHz) and 44.2% (3.35–5.25 GHz), respectively. Figure 8 also shows the measured LHCP gain, and peak gain of 5.66 dBic was achieved at 4.8 GHz. The measured 3 dB ARBW laid within the −10 dB reflection bandwidth; therefore, the entire bandwidth can be utilized for CP applications. Furthermore, good agreement was found between the simulated and measured results in Figure 7 and Figure 8, except for some discrepancies which can be attributed to fabrication tolerances.

**Figure 9:**
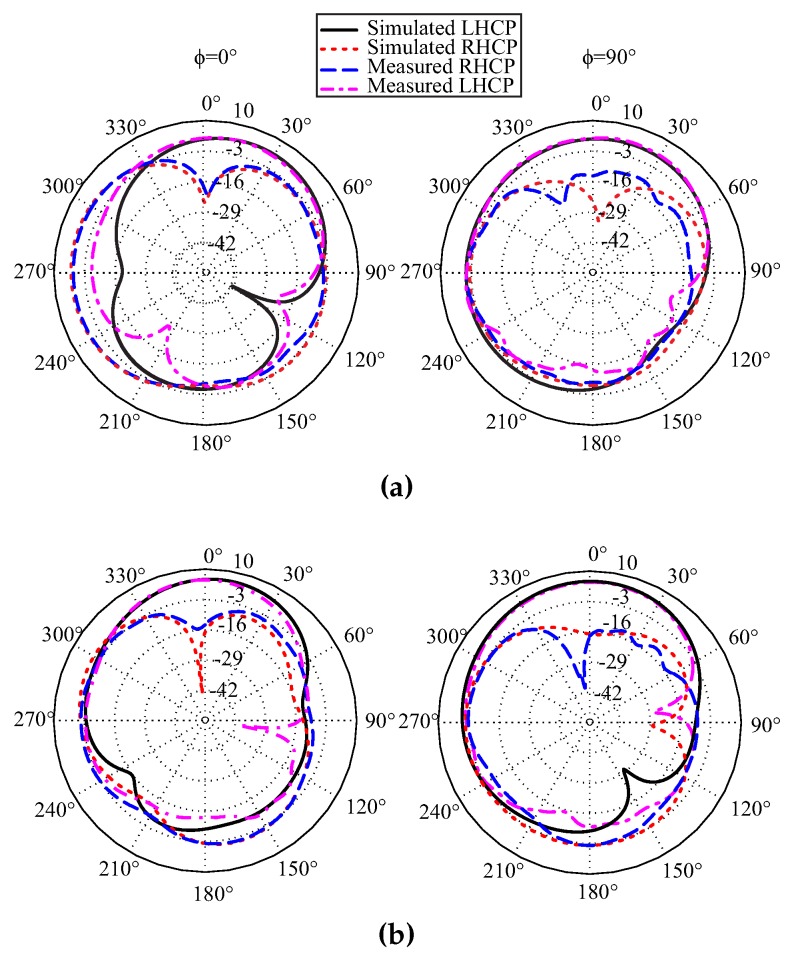

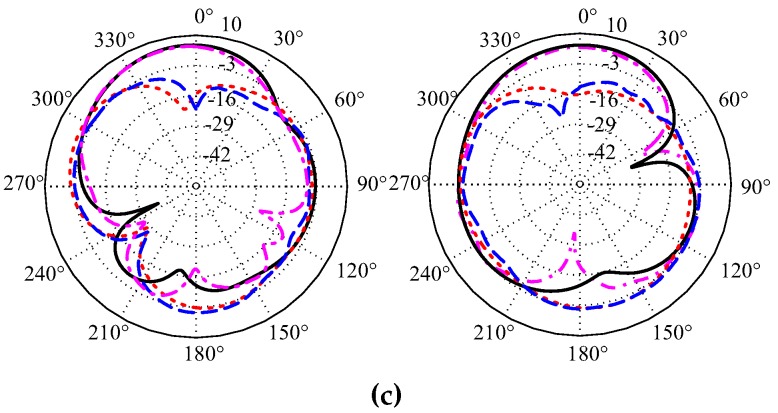
Simulated and measured radiation patterns of the proposed antenna: (**a**) 3.5 GHz; (**b**) 4.25 GHz; and (**c**) 5 GHz. RHCP: Right-handed circular polarization.

The measured 3 dB ARBW covers frequency bands of a number of applications, including WiMAX (3.5 GHz), radio altimeters (4.2–4.4 GHz), and different WLAN channels (4.9–5.15 GHz). Figure 9 shows the radiation patterns of the proposed antenna on the xz-plane (ϕ = 0${}^{{}^{\circ}}$) and yz-plane (ϕ = 90${}^{{}^{\circ}}$) at 3.5 GHz, 4.25 GHz, and 5 GHz. At all frequencies, the measured LHCP gain was higher than RHCP by more than 19.5 dB on the xz-plane and more than 16 dB on the yz-plane in the broadside direction.

Finally, a performance comparison of the proposed work is presented in relation to earlier published works [7,8,9,10,11]. This comparison is shown in Table 2. It can be seen that the proposed work has a wider −10 dB reflection bandwidth and a 3 dB ARBW along with more compact dimensions as compared to the other DRAs [7,8,9]. Another work [10] utilizes a modified microstrip feed to excite a cubic DR, and has dimensions of 0.44 × 0.44 × 0.21 $\lambda_{0}^{3}$ ($\lambda_{0}$ corresponds to the center frequency of the 3 dB ARBW). In comparison, the proposed work has larger dimensions of 0.66 × 0.83 × 0.39 $\lambda_{0}^{3}$ but possesses a wider −10 dB reflection bandwidth, a broader 3 dB ARBW, and a higher peak gain. Compared with one study [11], the proposed work offers superior performance in terms of the −10 dB reflection bandwidth and 3 dB ARBW. Therefore, it is concluded that the proposed work demonstrates good performance overall in terms of the −10 dB reflection bandwidth and 3 dB ARBW as well as size and peak gain.

## 5. Conclusions

A simple broadband CP DRA with vertical-strip excitation is presented. The antenna is designed with a partial ground plane. A fair performance comparison between the proposed and rectangular DRs is carried out. It is concluded that the proposed DR, due to its shape, combines multiple orthogonal modes to produce a broad 3 dB ARBW. The proposed antenna is evaluated after fabrication, achieving a measured −10 dB reflection bandwidth of 82.7% (2.44–5.88 GHz). The measured 3 dB ARBW of 44.2% (3.35–5.25 GHz) with a peak LHCP gain of 5.66 dBic lies within the −10 dB reflection bandwidth. Good agreement is found between the simulated and measured results. Radiation patterns were noted at different frequencies. On both planes, the purity of the measured LHCP gain is greater than 16 dB from RHCP in the broadside direction. In short, due to its broad 3 dB ARBW performance and simple feeding technique, the proposed antenna is a potential candidate for various CP applications.

## Figures and Tables

**Table 1:** Geometric parameters of the proposed antenna.

Parameters	Values	Parameters	Values
α	35.40${}^{{}^{\circ}}$	$d_{v}$	15.35 mm
a	20.20 mm	$f_{h}$	9.00 mm
b	24.80 mm	$f_{w}$	3.30 mm
c	13.20 mm	$f_{x}$	13.00 mm
d	25.60 mm	$f_{y}$	1.90 mm
e	7.60 mm	$f_{z}$	11.50 mm
g	2.60 mm	$g_{s}$	36.10 mm
h	1.52 mm	$g_{w}$	13.25 mm
m	14.90 mm	$g_{x}$	21.90 mm
$a_{x}$	2.00 mm	$g_{y}$	46.00 mm

**Table 2:** Comparison of the proposed work with earlier published work. Note that $\lambda_{0}$ represents the wavelength at the center frequency of the 3 dB ARBW. CP: circularly polarized; DRA: Dielectric resonator antenna.

Structure	Description	−10 dB Reflection Bandwidth (GHz)	3 dB ARBW (GHz)	Dimensions ($\lambda_{0}^{3}$)	Peak Gain (dBic)
[7]	CP DRA using a pixelated DR	2.62–3.63 (32.3%)	2.85–3.3 (14.6%)	1.44 × 1.44 × 0.34	6.13
[8]	CP DRA using a multi-circular-sector DR	1.88–2.58 (31.4%)	2.06-2.50 (19.3%)	0.84 × 0.84 × 0.39	7.65
[9]	CP DRA using a trapezoidal DR	2.88–4.04 (33.5%)	3.11–3.86 (21.5%)	1.16 × 1.16 × 0.44	8.39
[10]	CP DRA excited with a modified microstrip feed	2.62–3.63 (33.4%)	2.95–3.63 (20.6%)	0.44 × 0.44 × 0.21	1.51
[11]	CP DRA using a square DR with two unequal inclined slits	3.08–5.18 (50.8%)	3.08–4.43 (36%)	0.59 × 0.59 × 0.11	6
Proposed work	CP DRA using the proposed DR	2.44–5.88 (82.7%)	3.35–5.25 (44.2%)	0.66 × 0.83 × 0.39	5.66
